# Anemia prevalence at the time of pregnancy detection

**DOI:** 10.4274/tjod.06337

**Published:** 2017-09-30

**Authors:** Mustafa Öztürk, Özlem Öztürk, Mustafa Ulubay, Emre Karaşahin, Taner Özgürtaş, Müfit Yenen, Aytekin Aydın, Fahri Fıratlıgil, Serkan Bodur

**Affiliations:** 1 University of Health Sciences, Bakırköy Dr. Sadi Konuk Training and Research Hospital, Clinic of Obstetrics and Gynecology, İstanbul, Turkey; 2 Gülhane Training and Research Hospital, Clinic of Medical Biochemistry, Ankara, Turkey; 3 Gülhane Training and Research Hospital, Clinic of Obstetrics and Gynecology, Ankara, Turkey; 4 Kocaeli State Hospital, Clinic of Obstetrics and Gynecology, Kocaeli, Turkey

**Keywords:** Pregnancy, Anemia, prevalence

## Abstract

**Objective:**

Anemia in the first trimester of pregnancy is the situation as described by the World Health Organization when the level of hemoglobin (Hb) is less than 11 g in 100 cc of blood. The prevalence of this problem is 18% in developed countries, whereas it is between 35-75% in developing countries. In this study, we aimed to determine the prevalence of anemia at the time of pregnancy detection.

**Materials and Methods:**

A retrospective cross-sectional study was designed to determine the prevalence of anemia. A total of 5228 first trimester pregnant women were admitted to the study between 2012 and 2014. Hb levels of 11 to 9.5 g/dL, 9.5 to 8 g/dL, and less than 8 g/dL were considered as mild, moderate, and severe anemia, respectively.

**Results:**

We detected mild, modarate, and severe anemia at rates of 16.64%, 3.07%, and 0.28%, respectively, in our population. The overall prevalence of anemia at the time of detection of pregnancy was 20.0%.

**Conclusion:**

Anemia is a significant risk factor for maternal mortality in developing countries. The prevalence of anemia at the time of pregnancy detection was 20% and this rate is close to those indicated in developed countries.

## PRECIS:

The prevalence of anemia at the time of pregnancy detection was 20% and this is close to the rates of developed countries.

## INTRODUCTION

It has been demonstrated that anemia in pregnancy is one of the main health problems and affects the results of pregnancy negatively^(1)^. The prevalence of anemia is still in question in our country^(2)^.

Anemia can be classified as acquired or hereditary. Deficiency anemia (iron, folate, and vitamin B12), anemias depending on blood loss, chronic disease anemias, acquired hemolytic anemias, and aplastic anemia can be considered as acquired anemias, whereas sickle cell anemia, thalassemia, and Fanconi anemias are considered as hereditary anemias^(3)^. Iron-deficiency anemias (IDA) are responsible for more than half of all cases in all regions worldwide (where malaria is not an endemic). In pregnancy, the most frequently encountered anemia is IDA^(4)^.

Anemia in the 1^st^ and 3^rd^ trimester of pregnancy was defined by the Centers for Disease Control and Prevention in 1989 as hemoglobin (Hb) or hemotocrit less than 11 g/dL or 33%, respectively, and when the level of Hb or hemotocrit is less than 10.5 g/dL or 32%, respectively, in the 2^nd^ trimester of pregnancy^(1)^. According to the World Health Organization (WHO), anemia in pregnancy in any trimester is considered when the level of Hb in less than 11 g/dL^(4)^. This definition was made in 2001 and is still valid today. When the level of Hb is less than 7 g/dL during pregnancy, it is considered as severe anemia and medical treatment is required. It has been revealed that anemia observed in the first trimester of pregnancy enhances the possibility of premature birth and low birth weight, as well as low APGAR scores^(5,6)^.

In our study, we aimed to identify the prevalence of anemia in patients at the time of pregnancy detection.

## MATERIALS AND METHODS

This study is a retrospective cross sectional study that was designed to detect anemia prevalence of 5228 pregnant women who presented because of delayed menstrual periods and were diagnosed as being pregnant between 2012 and 2014. Hb levels of 11 to 9.5 g/dL, from 9.5 to 8 g/dL and less than 8 g/dL were considered as mild, moderate, and severe anemia, respectively^(7,8)^. Pregnants were classified into 4 groups according to their Hb levels as follows; group 1: severe anemia, group 2: moderate anemia, group 3 mild anemia, and group 4 as normal (Hb levels 11 g/dL or higher).

The complete blood count of the women was measured using an automated blood analyzer (Beckman-Coulter, USA). The three-year results were evaluated and classified according to their Hb levels. We did not report the risk factors and independent predictors of anemia. This study was approved by the Etimesgut Military Hospital Local Ethics Committee (approval number 8000-11-12) and all women who accepted to take part gave written informed consent before enrollment in the study.

### Statistical Analysis

The collected data were analyzed using the Statistical Package for Social Sciences version 14.0 (SPSS Inc., Chicago, USA). Continuous variables are expressed as mean ± standard deviation, whereas categorical variables are denoted as numbers or percentages where appropriate.

## RESULTS

**Figure 1:**
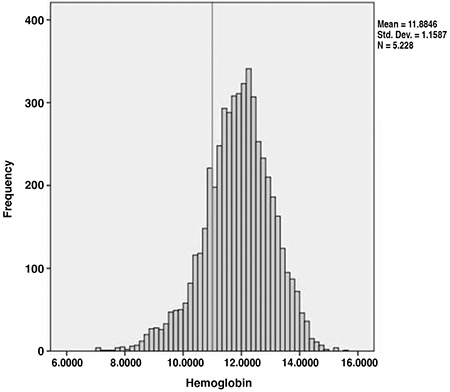
Distrubition of hemoglobin parameters of the pregnant women

Groups 1, 2, 3, and 4 comprised 15, 161, 870, and 4182 patients, respectively. The average age was 30.2±4.75 years and the average Hb was 11.8±1.15 g/dL in our population (Table 1) (Figure 1).

In groups 1, 2, 3, and 4, the average ages were 30.4±7.14, 30.2±5.42, 30.1±4.4, 30.2±4.7 years, respectively. The respective Hb levels in each group were 7.5±0.35, 9.0±0.34, 10.4±0.4, and 12.3±0.80 g/dL (Table 2).

In our population, when pregnancy was diagnosed, 16.64% (n=870) were considered as having mild anemia, 3.07% (n=161) had moderate anemia, and 0.28% (n=15) had severe anemia (Table 2). The overall anemia prevalence at the time of pregnancy diagnosis was 20.0% (n=1046).

**Figure 2:**
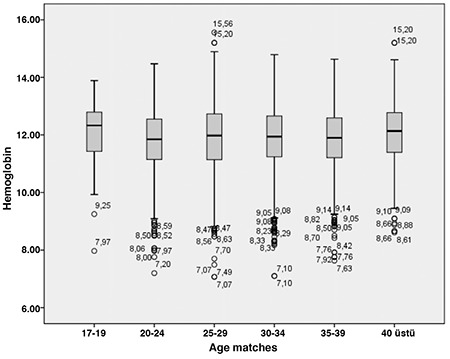
Distribution of pregnant women according to age and hemoglobin parameters

When the Hb levels were considered according to the age interval of the women, anemia prevalence was close to 20% in those aged 25-34 years (Table 3). Of the pregnant women, 7.3%, 21%, 20.5%, 19.9%, and 13.9% had anemia in the age groups 17-19 years, 20-24 years, 25-29 years, 30-34 years, and over 40 years, respectively (Table 3) (Figure 2).

## DISCUSSION

Anemia in pregnancy is a global public health problem. The prevalence is 18% in developed countries, whereas it is between 35-75% in developing countries^(9)^. In developing countries, it has been estimated that 460 million women of reproductive age are anaemic, 2/3 of whom are in Asia. It is known that prevalence of anemia in pregnancy is 42% worldwide, the lowest being 6% in North America and the highest is 75% in Gambia^(3)^. The prevalence of anemia in pregnancy is 25.1% in Europe, and around 24.1% in America^(10)^. In a study perfomed in China with 88149 pregnant women, the prevelance of anemia in the first trimester was determined as 22%^(5)^.

In our country, in a study performed in 2006 in which 586 pregnant women were included, the prevalence of anemia was determined as 74.1%^(2)^. In that study, the threshold Hb level for anemia was taken as 11 g/dL^(2)^. This level is close to that of underdeveloped countries. The prevalance of anemia in pregnancy in Turkey was determined as 40.2% by the WHO according to data observed before 2000^(3)^. In some Turkish studies, the prevalance of anemia during pregnancy was identified as 29.4% in Afyon, whereas it was 42.4% in Elazığ^(11,12)^. In a study by Karaoglu et al.^(13)^ with 823 pregnants, the prevalence of anemia was detected as 27.1%. The study was performed in Malatya and the Hb level was accepted as 11 g/dL^(13)^.

In the present study, the prevalence of anemia in pregnancy was determined as 20% in 5228 pregnant women in Ankara. In the study performed by Karaoglu et al.^(13)^, it was found that 0.48% of the pregnant women had severe anemia (under 8 g/dL); this rate was 0.28% in our study. This may be due to differences between patient populations in Ankara and Malatya.

When anemia prevalence was considered according to age intervals, Karaoglu et al.^(13)^ found that the rate was about 30% in pregnant women aged 30-39 years, wheras it was around 20% in our study for women aged 20-39 years. In a study by Pirinçci et al.^(12)^ that included data before 2001 in 465 pregnant women in Elazığ, it was shown that 42.4% (n=197) of patients had anemia (Hb levels below 11 g/dL); 44.8% of these were observed in the first trimester. Beside this, the authors reported the incidence of anemia as 59.4% for patients aged 19 years and below, 40.8% in the 20-29 years age group, 39.5% in the 30-39 years age group, and 25.0% in the >40 years age group^(12)^.

In our 2011-2015 data, the anemia prevalance was detected as 7.3% in patients aged 19 years and below, 26.1% in the 20-29 years age group, 24.8% in the 30-39 years age group, and 13.9% in the >40 years age group. In our study, the prevalence of anemia “at the time of pregnancy diagnosis” was determined as 20.1% because fertility is higher between the ages of 20-39 years. When our results are compared with those of Pirinçci et al.^(12)^, one might conclude that the prevalence of anemia decreased by half during this 10-year period. The effect of socioeconomic differences between Elazığ and Ankara and the presence of a more conscious pregnant population could also account for this difference. It was revealed that the incidence of anemia during pregnancy increased as pregnancy advanced (1.8% in the first trimester, 8% in the second trimester, and 27% in the third trimester)^(14)^.

In the studies mentioned above, the measurements for determining the prevalance of anemia were performed with regard to the number of weeks’ pregnancy. However, in our study, the prevalance of anemia was investigated in pregnant women in the first trimester only, and this could account for the differences between the rates observed in the indicated studies and our study.

Anemia in the first trimester of pregnancy increases the risk of preterm birth, small-for-gestational-age births, and intrauterine growth restriction^(15)^. For mothers, severe anemia is an important risk factor for morbidity and mortality in developing countries. The risk of operative birth and prolonged delivery increases in cases of severe anemia^(15)^.

### Study Limitations

This study has a limitation, the anemia prevalence in pregnancy “at the time of pregnancy diagnosis” was detected as 20% and this was close to the rates of developed countries. However, this rate could be related to the patient population of Ankara where the socioeconomic level is higher in comparison with other regions of our country.

## CONCLUSION

Anemia in pregnancy is a global public health problem and early diagnosis and treatment are both maternally and perinatally important. Detecting patients with anemia in the preconceptional period and/or delaying pregnancy until optimal Hb levels are reached will lower this rate.

## Figures and Tables

**Table 1:**
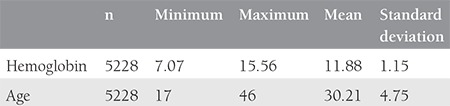
Age and hemoglobin parameters of the participants

**Table 2:**
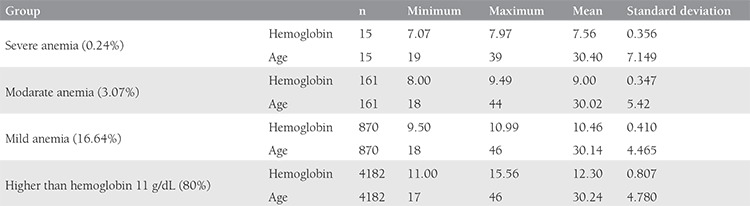
Age and hemoglobin parameters of the participants according severity groups

**Table 3:**
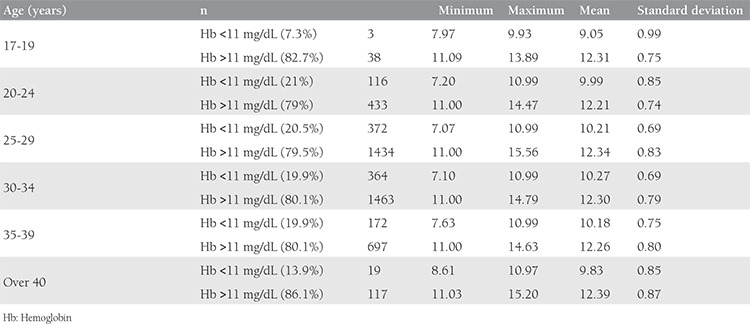
Distribution of pregnant women with anemia according to age ranges
